# Size- and time-dependent alteration in metabolic activities of human hepatic cytochrome P450 isozymes by gold nanoparticles via microsomal coincubations

**DOI:** 10.1186/1556-276X-9-642

**Published:** 2014-11-28

**Authors:** Meiling Ye, Ling Tang, Mengjun Luo, Jing Zhou, Bin Guo, Yangyuan Liu, Bo Chen

**Affiliations:** 1 Key Laboratory of Phytochemical R&D of Hunan Province and Key Laboratory of Chemical Biology and Traditional Chinese Medicine Research (Ministry of Education of China), Hunan Normal University, Changsha, 410081, China; 2 Yiyang Medical College, Yiyang 413000, China

**Keywords:** Gold nanoparticle, Cytochrome P450, Inhibition, Microsome, Incubation, Nanoprecipitation

## Abstract

Nano-sized particles are known to interfere with drug-metabolizing cytochrome P450 (CYP) enzymes, which can be anticipated to be a potential source of unintended adverse reactions, but the mechanisms underlying the inhibition are still not well understood. Herein we report a systematic investigation of the impacts of gold nanoparticles (AuNPs) on five major CYP isozymes under *in vitro* incubations of human liver microsomes (HLMs) with tannic acid (TA)-stabilized AuNPs in the size range of 5 to 100 nm. It is found that smaller AuNPs show more pronounced inhibitory effects on CYP2C9, CYP2C19, CYP2D6, and CYP3A4 in a dose-dependent manner, while 1A2 is the least susceptible to the AuNP inhibition. The size- and dose-dependent CYP-specific inhibition and the nonspecific drug-nanogold binding in the coincubation media can be significantly reduced by increasing the concentration ratio of microsomal proteins to AuNPs, probably via a noncompetitive mode. Remarkably, AuNPs are also found to exhibit a slow time-dependent inactivation of 2D6 and 3A4 in a *β*-nicotinamide adenine dinucleotide 2′-phosphate reduced tetrasodium salt hydrate (NADPH)-independent manner. During microsomal incubations, UV–vis spectroscopy, dynamic light scattering, and zeta-potential measurements were used to monitor the changes in particle properties under the miscellaneous AuNP/HLM/CYP dispersion system. An improved stability of AuNPs by mixing HLM with the gold nanocolloid reveals that the stabilization via AuNP-HLM interactions may occur on a faster time scale than the salt-induced nanoaggregation by incubation in phosphate buffer. The results suggest that the AuNP induced CYP inhibition can be partially attributed to its adhesion onto the enzymes to alter their structural conformations or onto the HLM membrane therefore impairing the integral membrane proteins. Additionally, AuNPs likely block the substrate pocket on the CYP surface, depending on both the particle characteristics and the structural diversity of the isozymes. These findings may represent additional mechanisms for the differential inhibitory effects arising from the coincubated AuNPs on the metabolic activities of the hepatic CYP isozymes.

## Background

Gold nanoparticles (AuNPs) have been broadly applied to various biomedical fields such as biosensing assays and clinical diagnoses, due to the unique physicochemical properties and biocompatibility which are distinctly different from those of their bulk counterparts at the nano-scale. Today, AuNPs are emerging as promising therapeutic agents for treatment of AIDS [1], tumor [2,3], and Parkinson's disease [4] and are being developed as a new form of drug carriers based on their own bioactivity, photo-thermal effect, and high drug-load capacity and membrane permeability. Additionally, more and more nanogold-containing products become readily available on the market, thus increasing the chance of exposure to and the health risks of engineered nanogold in food, cosmetics, and other consumer products through the skin, lungs, or gastrointestinal tract. As ultrasmall nanoparticles enter a biological system, their impact on and binding with biological components, especially tissue proteins are inevitable in both *in vitro* and *in vivo* biological exposure. It is now well established that, in biological fluids such as blood, the surface of nanoparticles becomes coated with a complex mixture of proteins to form the so-called ‘protein corona,’ which affects the NP-protein interactions and eventually determines their fate in the body. Extensive studies have been carried out on the NP-induced protein corona phenomenon, and the intrinsic characteristic of NPs (e.g., chemical composition, heterogeneity, size, shape and surface charge, curvature and functionalization) and the complex non-covalent forces occurring at the nano-bio interface (e.g., hydrogen bond, hydrophobic, electrostatic, Van der Waals, steric force, solvent and polymer bridging) are generally considered as the main factors controlling the surface adsorption and interactions [5,6]. However, Dominguez-Medina et al. hold an opposite view that formation of a protein layer is almost independent of NP shape [7] and ionic strength of a solution is an essential element in the formation of bovine serum albumin (BSA)-corona with NPs [8]. More recently, some corona proteins in serum have been determined by using quantitative proteomics analysis, but the adsorption mechanism has been left undiscovered in-depth [9-13]. Also, proteins may undergo conformation change and functionality missing upon association with NPs [14,15]. Compared to serum protein, an enzyme is a special type of protein molecules that can accelerate forming and breaking of chemical bonds required for essential biochemical reactions. In a polymerase chain reaction, NPs served as a new optimizer to DNA amplification by changing the equilibrium point of a DNA polymer concentration-activity curve and increasing the level of synthesis of DNA [16-18]. In addition, a few nanomaterial-enzyme conjugates have been reported to exhibit higher stability than the native enzymes [19]. On the other hand, an investigation of influences of carbon black nanoparticles on arylamine N-acetyltransferase [20] indicated that the NPs can change protein conformation and cause an irreversible binding to the major aromatic amine-metabolizing enzyme, thus leading to the loss of enzymatic acetylation activity toward carcinogenic aromatic amines.

Due to the pharmacological importance of xenobiotic-metabolizing enzymes and potential safety issues induced by NPs of toxicological concern, the influences of nanomaterials on these enzymes have gained an increasing interest from the general public and the scientific community. It is well known that the liver is the major tissue exposed to exogenous chemicals/encountered pollutants and the hepatic cytochrome P450 (CYPs) system localized in the endoplasmic reticulum is the largest and most important enzyme family for drug metabolism and plays a key role in detoxification and clearance of a wide range of xenobiotics and endogenous substances. As a special kind of exogenous materials exposed in bio-tissues, nanoparticles may be able to disrupt the biological functions of drug-metabolizing enzymes by a direct contact with CYPs. So, changes in NP-CYP interactions and NP-associated effects on CYP activities are to be expected. There is a few data available on the induction and inhibition of certain CYPs by several types of NPs, including metallic and polymeric nanomaterials. It has been shown that silver nanoparticles exhibit strong inhibitory effects on biotransformation activity of human CYPs especially 2C and 3A families [21-23] while AuNPs are approximately threefold weaker than AgNPs [24] with a similar metallic particle size. For investigating the NP-size effect on CYPs, Frohlich et al. [25] show that there is a size-dependent effect of carboxyl polystyrene NPs of 20 to 500 nm on metabolic activity of various CYPs. It was interesting to see that the inhibition potency was increasing with the decrease in particle size, i.e., the smallest polystyrene NPs (20 nm) have the highest effect on these enzymes while larger NPs (200 and 500 nm) showed 10% to 20% induction effects on 3A4 isoenzyme. It seems that the CYP enzymatic system is vulnerable to NPs. However, the diverseness in the influences of NPs on the CYP-mediated reactions is still poorly understood, and hence, an in-depth investigation of the NP-CYP interaction picture is required to identify the underlying factors affecting the NP-induced enzymatic alterations during the incubation.

Most recent studies focus on the NP-induced (negative) effects on enzymatic functions, but scarce reports of the potential impact of incubation matrix components on NPs have been published. Owing to the potentially complex interplays between NPs and biomolecules (e.g., corona) and ions (e.g., aggregation) in their environs, the mechanisms behind the disruption of CYPs and other cellular components (e.g., microsome membrane) in general are not necessarily obvious [26]. In the present study, we aimed to examine not only the effects of AuNPs on activity and kinetics of the five major human CYP450 isoenzymes (1A2, 2C9, 2C19, 2D6, 3A4) using the *in vitro* microsomal system but also the changes in properties of AuNPs during the incubation period. The particle characteristics, CYP types, and incubation variables (protein concentration, nonspecific binding, incubation buffer and time) were systematically studied to find out how NPs interfere with the metabolic process of xenobiotics.

## Methods

### Chemicals and reagents

Aqueous gold colloids with different nominal particle diameters of 5 to 100 nm were provided by Ted Pella (Redding, CA, USA) in mass concentration of 1.0 mg mL^-1^ (i.e., Au atomic molarity 5.08 mM). Each batch of AuNP stock suspensions (supplied in Milli-Q water) was characterized for particle size, mass concentration, and optical properties using a JEOL 1010 transmission electron microscope (TEM) (JEOL Ltd., Akishima-shi, Japan), a Thermo Fisher X Series 2 ICP-MS (Thermo Fisher Scientific, Waltham, MA, USA) and an Agilent 8453 UV-Visible Spectrometer (Agilent Technologies, Inc., Santa Clara, CA, USA), respectively [27] (Additional file 1: Table S1, Figure S1). Hydroxy tolbutamide, (*S*)-mephenytoin, and dehydro felodipine were provided by Toronto Research Chemicals (North York, Canada). Testosterone was purchased from Labor Dr. Ehrenstofer (Augsburg, Germany). 6*β*-hydroxytestosterone was purchased from Cerilliant (Cerilliant Corp., Round Rock, TX, USA). Phenacetin, acetaminophen, tolbutamide, 4-hydroxymephenytoin, felodipine, methyltestosterone, dextromethorphan, dextrorphan tartrate, BSA, and *β*-nicotinamide adenine dinucleotide 2′-phosphate reduced tetrasodium salt hydrate (NADPH) were purchased from Sigma-Aldrich (St. Louis, MO, USA).

Pooled human liver microsomes (HLMs) were supplied by Research Institute for Liver Diseases Co. Ltd. (Shanghai, China). Sodium chloride (NaCl), magnesium sulfate (MgSO_4_), disodium hydrogen phosphate (Na_2_HPO_4_), and sodium dihydrogen phosphate (NaH_2_PO_4_) were obtained from Sinopharm Chemical Reagent Co., Ltd. (Shanghai, China). Acetonitrile, methanol, and acetic acid were HPLC grade reagents from Fluka Co. (Buchs, Switzerland). All other reagents were of analytical grade. Deionized water (18.2 MΩ cm^-1^ resistivity) used throughout the study was prepared by a Milli-Q-Plus ultrapure water system (Millipore, Bedford, MA, USA).

### Preparation of metabolite standards, working solutions, and quality control samples

Phosphate buffer (PBS) was prepared from 100 mM mono- and dibasic sodium phosphate stock solutions and adjusted pH value to 7.4. Working solutions of HLMs were prepared by diluting pooled HLM (0.5 mL at 20 mg mL^-1^ protein) with PBS. Stock solutions of metabolites were prepared in acetonitrile/water (1:1, *v*/*v*) except for 6*β*-hydroxytestosterone which was dissolved in methanol and then diluted with acetonitrile/water (1:1, *v*/*v*) to obtain working standard solutions. The preparation of stock and working solutions of substrates was shown in Additional file 1: Table S2. Methyltestosterone used as the internal standard (IS) was prepared to 15.67 nM in acetonitrile, which also served as the stopping agent of the reaction incubations.

Fortified samples serving as matrix-matched calibration standards and quality control (QC) for method validation were prepared according to the previously published procedures [27,28]. The matrix effect (*ME*%) from coexisting NP-matrix constituents on quantification was estimated by comparing the signal peak area of the target analytes in neat water-acetonitrile (1:1, *v*/*v*) solution with that of the same amount of the analyte spiked into a post prepared supernatant from 200 μL of the NP-containing blank sample (postprecipitation spike). Method accuracy and precision was assessed by threefold determinations of each QC sample fortified at three different levels (5, 50, and 500 ng/g) in the individual matrices. The accuracy expressed as recovery (%) was calculated by the ratio of the measured concentration data to its nominal level. The mean recovery with its relative standard deviation (RSD) value from the sextuple measurements at each level was used to evaluate the precision. The benchtop stability of the test analytes in sample matrices during storage and processing were taken into account during the method validation. All of the measurements were conducted in triplicate, and the average value was compared with that obtained by an immediate injection of the freshly prepared control samples. Acceptance criteria for the assays were as follows: accuracy within 85% to 115% and precision of less than 15% at every concentration tested. All of the incubation samples, calibration standards, and control samples for both blanks (negative controls) and spikes (positive controls) were stored at -40°C (in the dark) until analysis.

### Microsomal incubation and nanoprecipitation extraction

Specific aspects of microsomal incubation condition for each assay (e.g., protein concentration, incubation time) are defined in Additional file 1: Table S2. In general, HLMs were mix with PBS, substrates, AuNPs, or a control solvent (deionized water) and warmed to 37°C for 3 min. CYP enzyme reaction was initiated by adding 40 μL NADPH (the final concentration was 1 mM) to a final incubation volume of 200 μL at 37°C for the period defined in Additional file 1: Table S2. Incubations were terminated by addition of 200 μL acetonitrile containing IS. Microsomes were stored at -80°C and thawed immediately before use. Frozen stocks of liver microsomes were used once with remaining material discarded. NADPH stock solution was made freshly before use.

The terminated incubation mixtures, as well as the matrix-matched calibration standards and QC samples, were subsequently processed by using a previously reported nanoprecipitation procedure [27] based on phase-separation induced nanoaggregation for rapid and complete separation of biocompatible NPs with minor modifications. Briefly, a salt mixture (20 mg NaCl plus 20 mg MgSO_4_) was added into the test sample (400 μL) to trigger nanoprecipitation. The mixture was vortexed vigorously for 1 min and centrifuged at 10,000 rpm for 5 min for the phase separation. Then a 10 μL aliquot of the supernatant (acetonitrile phase) was injected for direct liquid chromatography-mass spectrometry (LC-MS) analysis.

### LC-MS quantification of metabolites

LC-MS analyses were carried out on a QTRAP 4000 linear ion trap mass spectrometer (Applied Biosystems/MDS Sciex, Toronto, Canada) equipped with a TurboIonSpray electrospray ionization (ESI) source interfaced to a Prominence Series HPLC system consisting of LC-20 AD UFLC pumps, CTO-20A column oven, and SIL-20AHT autosampler (Shimadzu Corporation, Kyoto, Japan). Analyst 1.5 standard software from PE Sciex was used to control the instrument system and for data analysis.

LC separation was achieved using an Agilent Zorbax-SB-C18 column (150 × 4.6 mm i.d., 5 μm). Gradient elution at a flow rate of 0.5 ml/min was performed using acetonitrile (eluent A) and water containing 1% acetic acid (eluent B), with the gradient program as follows: 0 min, 10% A; 5 min, 70% A; 10 to 15 min, 100% A; and 15.1 to 18 min, 10% A. The run time was 18 min and the injection volume was 10 μL. Tandem MS detection was carried out using the positive-ion ESI source in the multiple reaction monitoring (MRM) mode for simultaneous quantitation of all the target metabolites and IS, with a precursor-to-product ion transition monitored for each analyte. All the source settings and instrument parameters were tuned to maximize generation of the protonated analyte molecule ([M + H]^+^) and to efficiently produce its characteristic fragment ions. Capillary voltage for positive electrospray was set at 5 kV, with a turbo ion spray interface source temperature at 550°C. The compound-dependent instrumental parameters of precursor-to-product ion transitions were optimized (Additional file 1: Table S1). A 100-ms dwell time with a 3-ms pause time was used for the all MRM transitions in one single retention time window.

### Dynamic characterization of AuNPs in microsomal incubation media

The same volume of 7- or 70-nm AuNPs and HLM were incubated for an hour. NPs in two concentrations (127, 304.8 μM) and HLM in five concentrations (0, 0.5, 5, 50, and 500 μg ml^-1^) were investigated; the plasmonic optical adsorption, hydrodynamic diameter, and Z-potential of AuNPs were read at regular intervals.

UV-visible adsorption spectrum of AuNPs was acquired over the wavelength range of 250 to 800 nm with UV-2450 spectrophotometer (Shimadzu, Japan) using quartz cuvettes with an optical path length of 0.5 cm at room temperature. Several metallic NPs, such as gold and silver, exhibit a characteristic absorbance maximum in visible range, namely surface plasmon resonance (SPR). This characteristic wavelength changes depending on size and surface alterations. SPR is sensitive to the surroundings of NPs at a molecular level; therefore, the changes of NPs in a close environment (such as biomolecular corona) can be investigated using this technique.

Dynamic light scattering (DLS) is a powerful tool for determining small changes in the size of NPs. The DLS and Z-potential experiments were performed on a Malvern Zetasizer Nano ZS (Malvern Instruments, Southborough, Massachusetts). Samples filtered followed by equilibration (typically 5 min) in a quartz cuvette to 37°C. The software was arranged with the specific parameters of refractive index and absorption coefficient of the material and the viscosity of the solvent. DLS allows determination of hydrodynamic diameter of colloidal particles and conjugates, that is the diameter of sphere with the same Brownian motion as the analyzed particle.

## Results and discussion

### Validation of nanoprecipitation-based LC-MS method for multi-metabolite quantification

To minimize the AuNP-associated matrix effects on liquid chromatography-mass spectrometric measurement of the enzymatic activities, we developed a novel nanoprecipitation approach for pretreating samples from the complex coincubation mixture. In a previous study, we have reported a facile salt-triggered precipitation method to efficiently remove water-soluble nanoparticles and extract analytes from complex NP-containing bio-samples into an organic phase for direct liquid chromatographic analysis [27]. In that work, the effects of solution properties on aggregation behavior of AuNPs in complex biomatrix-nanodispersion mixture were studied and a salt-induced nanoseparation procedure was developed as a new bio-sample pretreatment method for direct LC quantification. In general, tandem mass spectrometry detection offers superior sensitivity and selectivity that allows lowering the amount of microsomes in an incubation procedure, which can decrease nonspecific binding of inhibitor to phospholipids component [29] and enzymes [30] and thus reduce bias in kinetic constants [31]. However, the so-called matrix effect (i.e. ion suppression or enhancement) is a well known phenomenon and a major limitation in quantitative analysis with ESI mass spectrometry. In the present study, we first confirm whether the facile pretreatment protocol is suitable for LC-MS measurement. Herein we proposed this procedure as a new sample pretreatment for simultaneous LC-ESI-MS measurement of drug metabolic activities for cytochrome P450 enzymes in complex microsome-AuNP dispersion incubation mixture.

As shown in Table 1, the mean accuracy of hexaplicate measurements ranged from 88% to 112%, and the mean precision at the all QC levels were considered acceptable with RSDs of 1.2% to 11%. No AuNP-induced or biomatrix-related effects on ESI ionization were observed in microsomal incubation for LC-MS measurement of these metabolite analytes. The data demonstrated that the nanoprecipitation-based LC-MS multi-analyte quantitation using matrix-matched internal standard calibration provided satisfactory reliability with sufficient accuracy and precision.

**Table 1:** Summary of accuracy and precision of the analytical method for the six probe CYP metabolites

**Analyte**	**Enzyme**	**QC concentration (μM)**			**Mean accuracy (%)**			**Mean precision (RSD%)**		
		**Low**	**Mid**	**High**	**Low**	**Mid**	**High**	**Low**	**Mid**	**High**
Acetaminophen	CYP1A2	0.076	7.6	152.1	103	104	90	7.86	4.20	8.57
Hydroxy tolbutamide	CYP2C9	0.035	3.5	69.8	111	107	91	2.48	1.13	2.39
4-Hydroxymephenytoin	CYP2C19	0.043	4.3	85.38	103	102	112	6.35	2.73	3.23
Dextrorphan	CYP2D6	0.026	2.6	51.0	109	98	88	4.55	4.67	5.14
6*β*-Hydroxytestosterone	CYP3A4	0.033	3.3	65.7	98	110	93	10.28	4.75	7.27
Dehydro felodipine	CYP3A4	0.026	2.6	52.3	104	97	110	5.53	6.67	2.67

### Size- and dose-dependent alteration in metabolic activities of CYPs by AuNPs

**Figure 1:**
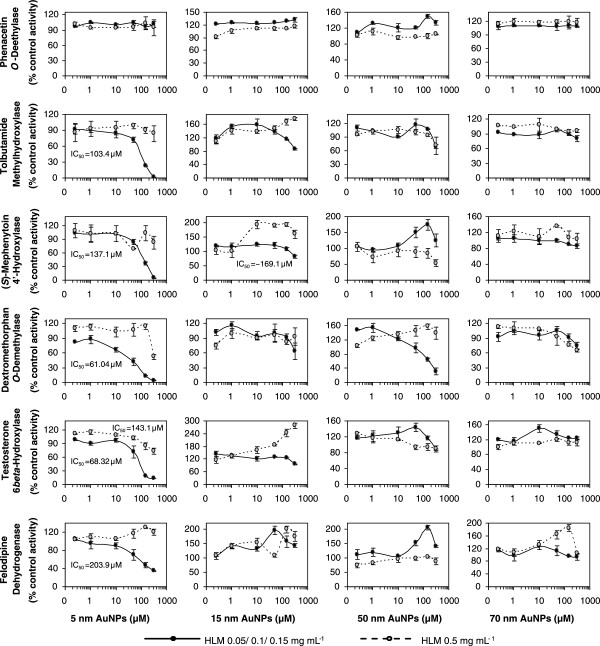
**The variation of AuNP-induced inhibition of six CYP450-selective activities at four different particle sizes.** The variation of AuNP-induced inhibition of six CYP450-selective activities at four different particle sizes with two microsomal protein concentrations. The monooxygenase activity was expressed as a percentage relative to the activity in the absence of AuNPs. Data points are mean values ± SD derived from duplicate incubations. *IC*_50_ were calculated with GraphPad Prism 5.

In this study we examine the influence of tannic acid-stabilized gold nanoparticles on a battery of CYP probe reactions conducted at near-*K*_m_ substrate concentration and different microsomal protein concentrations. AuNPs show no obvious effects on CYP450s at low particle concentration, but if the concentration is increased, CYP inhibition is observed (Figure 1). Under the normal incubation conditions where low HLM concentrations were used (as described in Additional file 1: Table S2), the inhibition effect depends on not only the AuNP concentration but also the particle size. In most cases, the percentage of control activity decreased with increasing NP concentration. For example, when the concentration of 5-nm AuNPs was increased to 317.5 μM, the remaining CYP isozyme activities were determined to be 1.94% for 2C9, 4.36% for 2C19, 3.53% for 2D6, 34.6% for 3A4 (felodipine dehydrogenase), and 13.25% for 3A4 (testosterone 6β-hydroxylase). The inhibitory effect was most pronounced on dextromethorphan *O*-demethylation (2D6) with the lowest *IC*_50_ value (61.04 μM), followed by testosterone *6β*-hydroxylation (3A4), tolbutamide methylhydroxylation (2C9), (*s*)-mephenytoin 4′-hydroxylation (2C19), and felodipine dehydrogenation (3A4), with the corresponding *IC*_50_ values of 68.3, 103.4, 137.1, and 203.9 μM, respectively. However, at the same mass concentration, AuNPs with different sizes (5 to 70 nm) showed different effects on CYPs. Smaller particles, particularly 5-nm AuNPs, showed pronounced inhibitory effects on CYP2C9, CYP2C19, CYP2D6, and CYP3A4, while 100-nm AuNPs caused minimal effect on these six CYP450-selective activities. The smallest effect was observed on phenacetin O-deethylase (1A2), even 5 nm at concentration above 300 μM caused a less than 10% inhibition of this enzyme. The result accords closely with earlier reports, where CYP1A2 was found to be much less susceptible to inhibition by small AuNPs [24], polystystyrene NPs [25], or nanosilver particles [23]. Although it appeared that not all CYPs were equally affected, it is noticed that the possibility of AuNP-drug interaction and caution about the potential adverse effects be taken into account when evaluating the *in vivo* relevance of observed *in vitro* inhibition for xenobiotics, in that approximately 85% of drugs in current use are primarily eliminated by these microsomal P450-catalyzed oxidations in humans [32]. Interestingly, the size- and dose-dependent effects of AuNPs can be reduced by increasing the microsomal protein concentration. As shown in Figure 1, when HLM concentration increased to 0.5 mg/mL, its sensitivity to AuNP-induced isoenzymatic inhibition was quite different. For 5-nm AuNPs, the degree of inhibition was apparently counteracted, thereby maintaining relatively high activities (more than 75%) even under 100 μM AuNPs. Fifteen-nanometer particles exhibited a slightly activatory effect, and the complex effects of large-sized AuNPs on enzymatic activities were reduced.

**Figure 2:**
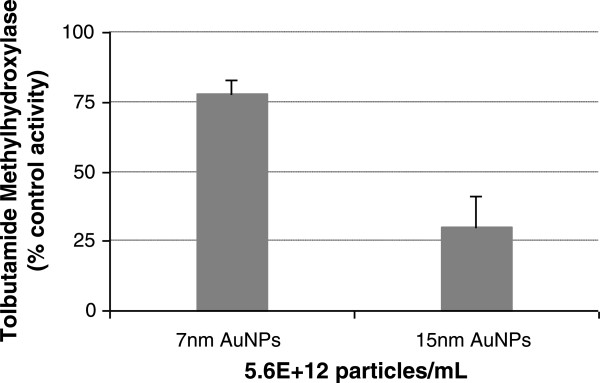
**AuNP-induced disruption of CYP2C9 activity with different particle size (7-nm and 15-nm) at the same particle concentration.** Data are mean values ± SD (*n* = 3) and are expressed as a percentage of the CYP activity without AuNPs.

In view of the huge variation in particle number between NPs of different sizes at the fixed mass concentration [27], a further study was designed to compare the AuNP-induced effect on CYP activities at the similar nanoparticle concentration. Figure 2 displayed the size-dependent inhibitory effect on tolbutamide methyl hydroxylation (CYP2C9) by 7-nm and 15-nm AuNPs at a particle concentration of 5.6 × 10^12^ particles/mL. The degree of inhibition by 15-nm AuNPs (70%) was observed to be about 3.4 times that of 7-nm AuNPs (21%). At fixed number concentration, increasing the particle radius leads to a higher mass concentration and larger NP surface area. As expected, the inhibition efficiency of 15-nm NPs is much higher than that of 7-nm NPs, owing to the larger surface area (ca. four- to fivefold) provided by the 15-nm NPs at the same particle concentration. This good agreement with the experimental results indicated that the nanogold inhibition of CYP-selective activities is highly dependent on the AuNP/protein ratio, probably via a typical noncompetitive inhibition mechanism anticipated from the protein (or NP surface area) dependence of the inhibition degree [23]. It is noted that surface modification of NPs may possess the capability of specifically regulating protein conformation and enzymatic function [33]. Tannic acid-coated AuNPs were used because they are more stable at high particle concentrations and low residual synthetic reactants in the final BioPure product of gold colloidal solutions [27]. As a multidentate capping agent, tannic acid was also reported to act as an inhibitor of several CYP-catalyzed oxidations in liver microsomes, especially inhibiting human CYP1A2 to the greatest extent [34]. However, this result was inconsistent with our findings. While a tannic-enzyme interaction makes up some of the CYP inhibitions occurring, there are likely to be many other pristine complicated NP-related interactions during the coincubation in that many CYP reactions could also be inhibited by citrate-capped AuNPs [24,26] or in a size-dependent manner and that CYP1A2 is found to be more undisturbed.

### Nonspecific adsorption of metabolites on AuNPs

The above *in vitro* incubation mixture is a complex miscellaneous HLM-NP dispersion system, and AuNPs will inevitably interact with the microsomal components (e.g., proteins phospholipids), cofactor (NADPH), substrates, and metabolites as well as buffering agents once involved in the incubation media. Most of all, there has been increasing evidence that NP-protein interactions play a crucial role in NP-induced conformational changes and enzymatic function impairments of the adsorbed enzymes [20-24]. However, besides the direct correlation between a loss of CYP activity and the metabolite formation measured, nonspecific adsorption of probe substrate or/and metabolite to AuNPs will also result in an overestimation of the enzymatic inhibition, and thus, the measured signal intensity for the marker metabolite in the incubation may not be truly reflective of the concentration available to be measured by the LC-MS instrument. There is no significant change in the concentration of probe substrates under the incubation conditions, indicating a low NP binding ratio of these compounds at high dose (5 to 120 μM). It is more important to note that nonspecific binding of drug metabolites onto AuNPs reduces the amount of the product transformed by the corresponding isoenzyme. Herein we focus on the impact and possibility of nonspecific adsorption of marker metabolites to AuNPs on the determination of enzymatic inhibition.

**Figure 3:**
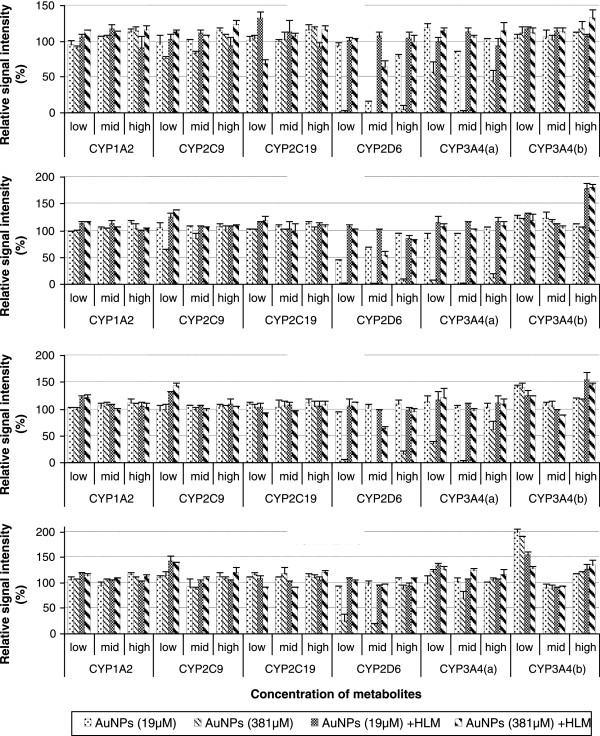
**Dose- and size-dependent nonspecific binding of AuNPs with CYP marker metabolites.** Six probe metabolites with standard solutions (low, medium, and high concentrations) were coincubated separately with 5, 7, 30, 70 nm AuNPs in PBS to a final volume of 200 μL at 37°C, in the absence or presence of 0.15 mg mL^-1^ HLM, and terminated the incubation after 30 min by adding 200 μL of acetonitrile containing IS. CYP3A4 (a) represents testosterone 6*β*-hydroxylase, and CYP3A4 (b) represents felodipine dehydrogenase. Data are mean values ± SD (*n* = 3) and are expressed as a percentage of QC samples of the metabolites incubated without AuNPs.

Figure 3 shows the average signal intensity of six CYP metabolites (at three QC levels) coincubated with AuNPs of different sizes (5, 7, 30, and 70 nm) at two NP concentrations (19 and 381 μM) without or with HLM, as a percent change relative to the data from the QC sample in the absence of any AuNPs (100%). When coincubated without HLM, AuNPs exhibited a strong dose- and size-dependent adsorption capacity for dextrorphan (2D6) and 6β-hydroxytestosterone (3A4), while almost no affinity to deethylphenacetin (1A2), 4′-hydroxymephenytoin (2C19), and dehydro felodipine (3A4). For example, low NP concentration (19 μM) of 5- and 7-nm AuNPs showed a moderate degree of attachment (>30%) but AuNPs of 30- and 70-nm had no adsorption (90% to 105%) to dextrorphan and 6β-hydroxytestosterone. Remarkably, high concentration (381 μM) of AuNPs caused more severe nonspecific binding problems associated with the two metabolites, indicating a dramatic reduction in signal intensity with relative percentage recoveries at 0% to 40%, 3% to 20%, 5% to 60%, and 20% to 101% by 5-, 7-, 30-, and 70-nm AuNPs, respectively. Fortunately, much of the nonspecific binding effects could be significantly reduced by incubating with HLMs, regardless of the NP concentration or size. In the presence of HLM, even high concentration of small-sized AuNPs could adsorb about 40% of dextrorphan (2D6) or 30% hydroxymephenytoin (2C19) at low metabolite concentration. Thus, despite the existence of a considerable degree of metabolite adsorption on AuNPs, the presence of HLMs in the typical *in vitro* probe reactions substantially reduces the adsorption effects on the measurement of catalytic activity. Given the extremely low residual activity observed for CYP2D6 (3.5%) and CYP2C19 (4.3%), far below 60% and 70%, respectively, under the inhibition experiments by coincubating with 5-nm AuNPs (Figure 1), this result suggested there must still exist additional mechanisms contributing to the degradation of CYP activity via enzymatic inhibition.

### Time-dependent AuNP-induced CYP inhibition

It was reported that NPs have also been implicated as irreversible inactivators of several enzymes which have crucial catalytic activities, such as chymotrypsin [35], acetylcholinesterase [36], and arylamine N-acetyltransferase [20]. Broadly, irreversibility does not necessarily mean the enzymatic activity never restores, only that the enzyme gets disfunctioned over an extended period of time. In general, time-dependent inactivations refer to any phenomenon resulting in a reduction of enzyme activity with preincubation time, which covers slow- and tight-binding inhibitions. Compared with reversible inhibition, time-dependent inhibition resulting from irreversible covalent or quasi-irreversible binding of a reactive intermediate or slow tight-binding inhibitor to the enzyme is often viewed as more serious, since the activity of the inactivated enzyme will not be returned until the new enzyme is resynthesized [37]. Moreover, the time-dependent loss of enzyme function has become more frequent in P450-catalyzed reactions, and more attention is now being increasingly focused on interpreting the consequences, either from a clinical pharmacokinetic or toxicological perspective [38].

**Figure 4:**
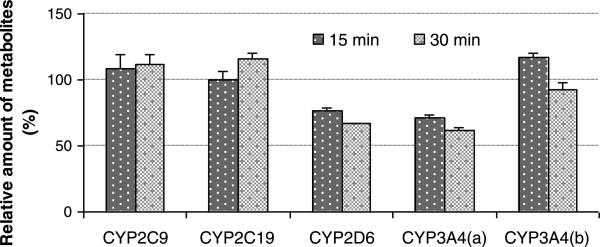
**Time-dependent changes in the specific activity of CYPs exposed to AuNPs for different preincubation periods.** The preincubation was performed in a PBS buffer (100 mM, pH 7.4) at 37°C, containing 2 mg/mL HLM, 100 μM 5-nm AuNPs with or without 1 mM NADPH, followed by a tenfold dilution of the preincubation reaction mixture into the second incubation for measuring the residual CYP activity toward individual probe substrates. The CYP preincubations for 0 min were used as the control incubations (100%), and error bars indicate the SD values.

In this study, we are seeking to examine whether AuNPs can behave as a time-dependent inactivator of the human CYP isoforms, by using a typical two-step incubation protocol which involves an inactivation preincubation for different time periods in the presence of AuNPs and CYPs (with or without NADPH) followed by residual CYP activity measurement by dilution into a secondary incubation containing the CYP isoform-specific substrate plus NADPH. Figure 4 shows the preincubation time-dependent changes in the specific activity of CYPs exposed to 5-nm AuNPs during the preincubations. There were no inhibitory effects of preincubation time on the marker reactions for 2C9, 2C19, and 3A4(b) in the presence of AuNPs. Notably, an addition of 5-nm AuNPs caused a time-dependent decrease in the formation of dextrorphan (2D6) and 6β-hydroxytestosterone (3A4(a)), leading to a significant reduction of activity to 74% to 76% and 62% to 66%, after 15 and 30 min of preincubation, respectively. However, a further investigation of the inhibitory effect on CYPs via interaction with AuNPs was also revealed that the time-dependent inhibitions remained unchanged in the presence and absence of NADPH. NADPH is a necessary cofactor in CYP-mediated biotransformation of xenobiotics. This is quite different from typical mechanism-based P450 inhibitions [37,38], a well-known form of irreversible enzyme inhibition that is characterized as displaying time-, concentration-, and NADPH-dependent inactivation due to formation of a (quasi-)irreversible complex of CYP with an inhibitor intermediate/metabolite via the normal catalysis reaction.

Based on the observed dose-dependent inhibition results (Figure 1), combined with the above time-dependent inactivation in a NADPH-independent manner (Figure 4), small AuNPs can act as reversible inhibitors of CYP2C9 and CYP2C19 and also exhibit a slow irreversible inhibition of CYP2D6 and CYP3A4 over time. Given that the size of the AuNPs (above 5 nm in diameter) far exceeds the dimensions of the largest heme catalytic pockets of CYP450s [39] for diverse substrate ligands (with molecular weights typically below 500 Da), even with a greater increase in active site cavity volume upon flexible ligand binding [40], the NP-induced inhibition appears to be on the enzyme surface rather than at its catalytic site [21,23]. Additionally, as anticipated from the microsomal protein-to-NP ratio dependence of the degree of inhibition (Figure 1), the nature of the reversible inhibitions was further identified to be noncompetitive. Similar results were also observed in recent research findings on NP-mediated CYP inhibitions [23,25], indicating that biophysicochemical disruption from nonspecific NP binding at surface sites through non-covalent interactions would be supported as an underlying mechanism for the reversible enzyme inhibitions. Exceptionally, it was reported that surface-functionalized carbon nanotubes were capable of recognizing and site-specifically binding to chymotrypsin's catalytic site and thus competitively inhibiting the enzymatic activity [41]. As for cytochrome P450 enzymes, a variety of irreversible inhibitors proved to be selective mechanism-based inactivators (suicide substrates) through their chemical modifications of the CYP heme and/or heme-thiolate proteins. Recently, most nanosized particles have been reported to inhibit a CYP activity due to direct electrostatic interactions and/or microenvironment changes in the surrounding ionic strength [21-26], while no data on time-dependent inactivation have been reported so far. In our study, tannic acid-decorated AuNPs were demonstrated to inhibit CYP2D6 and CYP3A4 in a time-dependent but NADPH-independent manner, indicating a conformational change in the enzyme during the slow and tight-binding and thereby affecting its biotransformation activity. A similar interpretation was also supported from previous observations with small (around 10-nm size) amino acid-functionalized AuNPs and carbon black NPs, which slowly inactivated the enzymatic activities of chymotrypsin [35] and N-acetyltransferase [20], respectively, through a two-stage mechanism involving a fast reversible inhibition (electrostatic binding) and a subsequent slower irreversible denaturation process over time.

### Stability of AuNPs during microsomal incubation

In some ways, the NP dispersion in such a complex biological medium may cause a shift in the particle size distribution or even some aggregation, which can significantly distort the available NP dose and consequently misinterpret any results obtained for NP interactions with living systems. Although the tannic acid-stabilized AuNPs are highly monodispersed with sufficient electrostatic repulsion to maintain the colloidal stability in aqueous solution (Additional file 1: Figure S1), it has been reported to be highly dependent upon the surrounding solution properties particularly the electrolyte concentration [27]. It is reasonable to hypothesize that any loss of P450 activity observed in this high-ionic-strength matrix and protein-rich biological environment could possibly be due to unexpected changes in NP's unique morphology, physicochemical nature, and bio-reactivity toward enzymes. For the purpose of gaining new insights into the mechanisms by which AuNPs inactivate CYPs, this work further investigated the influence of coincubation medium and experimental condition on the stability of AuNPs during the normal microsomal incubations.

**Figure 5:**
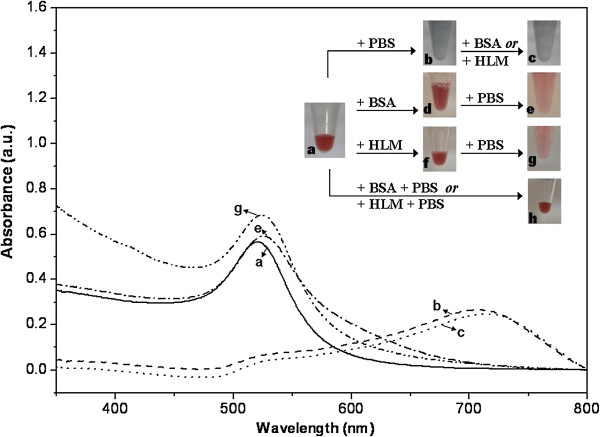
**Alteration in the UV–vis spectrum and appearance of AuNPs by mixing with PBS and proteins.** Alteration in the original UV–vis absorption spectrum and appearance of tannic acid-stabilized AuNPs (a: 30-nm, 127 μM) after mixing with phosphate buffered saline (PBS, pH 7.4, 100 mM) and 0.15 mg/mL protein (BSA or HLM) under different adding order of agents. Inset shows the flow chart of this procedure and digital photos of AuNPs solution in every step.

In our study, aggregation of AuNPs was observed in 0.1 M PBS (pH 7.4), but the presence of microsome or BSA in the *in vitro* incubation media could effectively prevent the aggregation. It is clear that a significant color change from red to blue and red shifts with two plasmon bands were observed after adding the PBS buffer solution into pure aqueous colloidal gold nanoparticles. For 30-nm AuNPs, the intensity of the highest absorbance (*λ*_max_) at 521 nm (in water as control) decreased dramatically (Figure 5a) and a new peak appeared around 707 nm (Figure 5b), indicating occurrence of the electrolyte-induced nanoaggregation consistent with our previous literature [27]. The interactions between tannic acid-coated AuNPs and cations in the buffer solution (e.g., Na^+^) could cause a decrease of AuNP optical density and localized surface plasmon resonance. Once the NP aggregates are initialized, subsequent nanoprecipitation will occur and their monodispersion status cannot be reversed by adding proteins (Figure 5c) because the tannic acid anions are inclined to form complexes with the buffer counter cations. However, the addition of BSA or HLM before mixing with PBS dramatically enhances the colloidal stability (Figure 5e,g). This proved that colloidal stability was also found even if BSA (or HLM) and PBS were simultaneously added into the aqueous gold colloids (Figure 5h), suggesting that AuNP stabilization via protein (or biomembrane) adsorption onto the NP surface occurs on a faster time scale than the salt-induced particle aggregation and precipitation. This observation was similar with the previous work suggesting that biomacromolecules can perform as a protection layer via NP surface adsorption to prevent particle aggregation and sedimentation under harsh conditions [8]. In the present CYP assays, HLM, PBS, and substrates were mixed firstly, and the nanodispersion kept pale red after the coincubation of AuNPs and NADPH, which means the protection against colloidal aggregation offered by HLMs can maintain a good dispersed state of tannic-coated AuNPs in saline solution under our typical experimental conditions.

**Figure 6:**
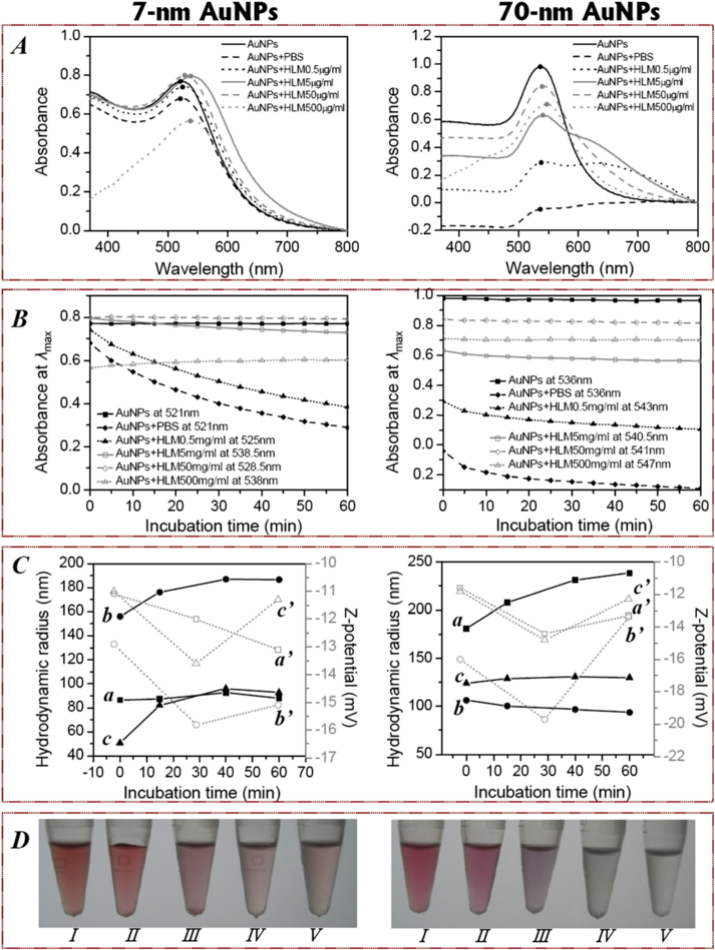
**Time-, size-, and concentration-dependent changes in particle properties during AuNP-HLM coincubations.** UV–vis extinction spectra **(A)**, variation of UV–vis absorbance value at *λ*_max_**(B)**, hydrodynamic diameter and Z-potential **(C)** and photograph of solutions **(D)** of 7 nm (left) and 70 nm (right) AuNPs in the presence of an equal volume of HLM for increasing protein concentrations during a coincubation period of 60 min. **(A)** The UV–vis spectra were acquired after mixing for 10 min, and the dots highlight the maximum adsorption of each spectrum. **(B)** UV–vis absorbance at *λ*_max_ as a function of incubation time extracted from equilibrated spectra in **(A)**. **(C)** Hydrodynamic diameter of AuNPs when mixed with 0.5 (a), 5 (b), and 50 μg mL^-1^ HLM (c); Z-potential of AuNPs when mixed with 0.5 (a’), 5 (b’), and 50 μg mL^-1^ HLM (c’). **(D)** A digital picture of AuNP solutions after 60 min of incubation with 500 (I), 50 (II), 5 (III), 0.5 (IV), and 0 μg mL^-1^ HLM (V) in the presence of 100 mM PBS.

To obtain more details on interaction between AuNPs and biological components, we monitored the dynamic changes of plasmonic optical adsorption, along with hydrodynamic diameter and Z-potential of AuNPs of different sizes at two NP concentrations during microsomal incubation under five HLM protein concentrations. The main research results for the higher NP concentration were described in Figure 6, and the lower concentration of AuNPs was observed to follow similar trends but with weaker absorbance intensity (data not shown). Figure 6A shows the extinction spectra of AuNP solutions after 10 min of equilibrium for different HLM concentrations in 100 mM PBS, and these spectra were also acquired as a function of incubation time after initial mixing as shown in Figure 6B. The time-dependent reduction in electron density of AuNPs in the buffer solution was strongly dependent on the presence of HLM and the NP size. After mixing with 100 mM PBS, the UV–vis extinction of the surface plasmon resonance at *λ*_max_ decreases significantly with time for the first 60 min of incubation, but the addition of HLM above 5 μg mL^-1^ protein could effectively stop the salt-induced aggregation process (Figure 6D). However, further increasing the HLM protein to 500 μg mL^-1^ reduced the surface plasmon absorbance, accompanied by broadening and distortion of the plasmon band in the range of 200 to 500 nm (Figure 6A). Compared with a gradual decrease in optical density of 7-nm AuNPs during the first 60 min, a sharp decline in the absorbance was observed for 70-nm AuNPs within the initial 5 min (Figure 6B), indicating the particle aggregation of larger AuNPs may occur on a much faster timescale than the smaller size particles in the early stage.

We also used the DLS technique to monitor the number of average particle diameter and zeta-potential under real incubation conditions, which provided an orthogonal comparison to the spectroscopic methods for the direct physical characterization of molecular conjugation to AuNPs. Table 2 summarizes the changes of UV–vis absorbance spectra, size, and *Z*-potential before and after incubation with HLMs. For 7-nm AuNPs, the effective diameter sharply increased from 10 nm to 155 nm (0 min) and then to 186 nm (60 min) after addition of 5 μg mL^-1^ HLM with PBS, and it increased to 50 nm (0 min) and then to 93 nm (60 min) by incubating with 50 μg mL^-1^ HLM (in PBS). The NP size remained almost unchanged (at around 86 nm) during the incubation period under 0.5 μg mL^-1^ HLM (Figure 6C). Although the NP zeta-potential displays an obvious variation during the microsomal incubation, it increased toward neutral values with offsets of 8 to 10 mV (Table 2). The surface potential of tannic acid (TA)-stabilized AuNPs becomes less negative, which could imply that HLMs can be bound to the NP surface since microsomes are just slightly negatively charged in pH 7.4 PBS buffer (-10.7 mV). It is important to establish whether the increase in the mean effective hydrodynamic radius is indeed due to particle aggregation or molecular adsorption. As for 70-nm AuNPs, the time-dependent increase in the particle diameter (from 78 to 181 then to 239 nm) occurs together with a decrease in the extinction (from 0.98 to 0.29 then to 0.11 a.u.) at *λ*_max_ under low HLM concentration (0.5 μg mL^-1^), corresponding to the formation of a small number of aggregates. While 7-nm AuNPs exhibit the maximum red shift of *λ*_max_ (17.5 nm) and maximum *D*_h_ increment (145.3 nm) when mixed with HLM at the intermediate concentration (5 μg mL^-1^), further confirming the possibility of strong adsorption and interactions between the microsomal membrane components and AuNP surface. In the case of higher HLM content (to 500 μg mL^-1^), the UV–vis spectrum is distorted in the range of 200 to 500 nm and yields a remarkably red shifted absorption band at *λ*_max_ probably because too many excess interacting biomolecules decrease the surface plasmon resonance of the particles.

**Table 2:** **Summary of the alteration of *λ***_**max**_**, *D***_**h**_**, and *ζ *** **by AuNP-HLM interactions**

**Test sample**	***λ***_**max **_**(nm)**	**Abs (a.u.)**	***D***_**h **_**(nm)**	***ζ *** **(mV)**
7-nm AuNPs				
in water	521	0.770	10.11	-21.3
in PBS (without HLM)	521	0.681	/	/
in 0.5 mg/ml HLM	525	0.741	85.74	-11.1
in 5 mg/ml HLM	539	0.795	155.34	-12.9
in 50 mg/ml HLM	529	0.801	49.72	-11.0
in 500 mg/ml HLM	538	0.566	/	/
70-nm AuNPs				
in water	536	0.981	78.22	-36.1
in PBS	537	0.012	/	/
in 0.5 mg/ml HLM	545	0.294	180.9	-26.4
in 5 mg/ml HLM	541	0.632	106.3	-30.8
in 50 mg/ml HLM	542	0.842	123.8	-26.6
in 500 mg/ml HLM	547	0.711	/	/
HLM in PBS buffer	/	/	112.2	-10.7
BSA in PBS buffer	/	/	7.93	-8.1

The alteration of absorbance (Abs) at λmax, along with hydrodynamic diameter (*D*_h_) and *Z*-potential (*ζ*) of AuNPs after initial mixing (at 0 min) with the same volume of HLM solution prepared in 100 mM PBS.

Unlike HLM, although bovine serum albumin (BSA) could slow down the NP aggregation rate in buffer as mentioned above, no effects of BSA on *λ*_max_ red shift of the tannic acid-stabilized AuNPs were observed (Additional file 1: Figure S2). A slight reduction in the electron density of AuNPs was observed dependent on protein level and NP size, suggesting a much weaker protein-particle interaction other than NP size increase in the solution [26]. BSA is the most widely used globular protein molecule approximated as a triangular equilateral prism with dimensions of 8 × 8 × 3 nm^3^[7]. Despite its net negative surface charge under physiological conditions, BSA can also be adsorbed to negatively charged AuNPs via a complementary electrostatic interaction with positively charged lysine residues or by a cysteine thiol-gold (Au-S) contact. However in this work, the CYP450 isoenzymes are integral transmembrane proteins that are bound to sealed microsomal vesicles derived mainly from the human liver endoplasmic reticulum [32]. The microsomal biomembrane is composed of two layers of amphiphilic lipid molecules, and such phospholipid bilayer membrane plays a vital role in regulating the activity and function of the membrane-bound CYP450s. Our DLS measurements revealed that the hydrodynamic diameters of BSA and HLM in PBS are about 7.9 and 112.2 nm, respectively, and that BSA and HLM in PBS had similar net negative surface potentials around -10 mV (Table 2), consistent with previously reported data from BSA [42] and *in vitro* supersome/microsome-based incubation [26]. The recorded changes show an increase in the surface potential and NP size of approximately 5 to 10 mV and 30 to 150 nm, respectively. However, based on the typical dimensions of the native form of the microsomes, the small discrepancy between the size increase and the HLM dimensions may be due to a solvent hydration shell and the heterogeneous size distribution of the AuNP-HLM incubation mixture [7,26].

### Proposed mechanisms for AuNP-CYP interactions

**Figure 7:**
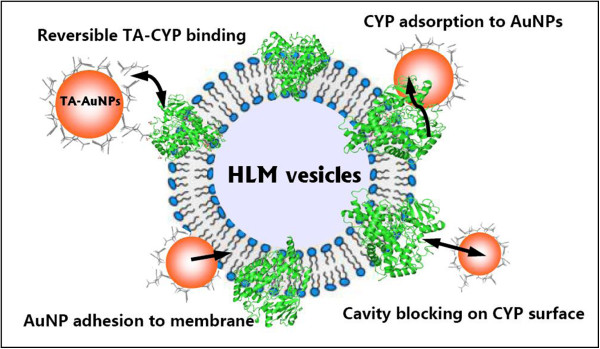
**Schematic illustration for the proposed interaction modes between TA-coated AuNPs and CYP isozymes.** Schematic illustration for the proposed interaction modes between tannic acid-coated AuNPs and CYP isozymes in the vesicular membrane structure of the microsomes.

The mammalian CYPs are N-terminally anchored to the endoplasmic reticulum membrane with their large cytoplasmic catalytic domains in the shape of a triangular prism [40]. Since the NP size is akin to proteins, phospholipids, and other biomacromolecular components of cell membranes, NPs can also disrupt the membrane topology or lipid bilayer structure and thereby influence the function of the membrane-bound enzymes. Various mechanisms proposed to account for NP-induced inhibition of enzymes can be generalized as enzyme conformation change, structure disruption, or/and function impairment through NP-protein interactions [20,21,23,26,35,42]. However, the exact mechanism of how NPs affect CYP enzymatic functions is inconsistent or still remains unclear. Sereemaspun et al. [24] considered NPs forming mixed micelles with microsomal membrane leading to change in membrane integrity and inactivation of enzyme. Lamb et al. [23] thought that particle inhibition may take place just at a surface site of an enzyme other than a catalyst site, and hence, physical disruption would be supported as a mechanism. Frohlich et al. [25] found that the highest effect of smallest polystyrene NPs was due to the hydrophobicity of NPs and the adsorption of the enzymes on them. Recently, Lu et al. [26] have proposed that the influence of AuNPs on CYP2B1-mediated stereoselective metabolism may be due to a combined effect of direct disruption of enzyme conformation and indirect alteration in ionic strength of microenvironment for co-factor production. In the present work, on the basis of the above in-depth investigations on the microsomal incubation variables by simultaneous measurement of CYP isozyme activities and monitoring of AuNP characters, it is possible to track the systematical changes during the AuNP-HLM coincubation and identify the potential underpinning interaction modes between tannic acid-coated AuNPs and CYP isozymes in the microsomal vesicle membrane (Figure 7).

First, CYP isozymes can adsorb on AuNPs to alter protein conformations and thus cause their functional impacts, with both stabilization (activation) and inhibition (deactivation) being possible. Soft (non-covalent and reversible) interactions between negatively charged NPs and proteins were reported to modify the tertiary structure of proteins and enzymatic activity in a size-dependent manner [42]. It is noticed that some strong interactions via electrostatic attraction, an Au-S linkage or/and irreversible modification are still occurring, which leads to structural changes and subsequent time-dependent enzyme inactivation [20]. However, our results showed that small AuNPs with a larger curvature gave a stronger inhibitory effect on CYPs, in contrast to the cases reported for lysozyme, ribonuclease A, and carbonic anhydrase onto silica NPs of different sizes [33], due to the larger number (total surface area) and higher mobility of smaller particles available for these membrane-bound proteins. Second, when CYPs are bound to the surface-coated AuNPs, their enzymatic activities may possibly be differentially regulated by the capping agent (tannic acid) at the NP-protein interface. Indeed, some chemically modified AuNPs can selectively inhibit or denature certain proteins through specific enzyme-inhibitor interactions [33,35]. As pointed out above, while TA showed an inhibitory effect on several CYPs especially CYP1A2 [34], our observation indicates that this effect is relatively weak and the TA-CYP binding is considered to be nonspecific or reversible. Third, the presence of the vesicular membrane structure can probably confer additional interplays between the TA-coated NPs and the microsomal surface including membrane adhesion and perturbation, thus impacting the function of the integral membrane proteins. It has been proposed that metallic NPs could be considered amphiphilic to fluidize and penetrate the microsomal membrane by forming mixed micelles with the biomembrane [24]. Furthermore, this permeation ability appears to be independent of the surface charge as both the NPs and microsomal membrane remain negatively charged [22], probably because the anionic NPs bearing an ordered amphiphilic surface structure can overcome the electrostatic repulsion [43]. Tannic acid is a type of polyphenol with weak acidity due to the numerous phenol groups in the structure. Based on the UV–vis and DLS measurements presented here, we currently cannot confirm the bilayer disruption or an ordered TA layer formed on the NP surface directly through the bulk characterizations. However, the TA-capped AuNPs exhibit a strong interaction with HLM membranes during the incubation. In particular, recent reports have provided evidence that the smaller size, TA-coated AuNPs distributes onto lipid bilayer more rapidly, due to the high affinity of TA to phospholipid membrane [44,45], further supporting the NP adhesion to the HLM membrane as a potential mechanism for CYP inhibition. Fourth, since the particle inhibition seems to be at a surface site [21,23,25], the AuNP-induced decrease of the CYP activity is likely to arise from steric hindrance in the catalytic cavity on the enzyme surface. Wang et al. [42] have supposed that if the time scale of the enzyme-NP interaction is comparable with that of the enzyme-substrate binding, a temporary blocking of the active site of enzyme may be expected and the NP acts as a competitor to the substrate. In a previous report, metallic NPs were assumed to bind competitively to the active site due to the hydrophobicity of the NPs and the heme environment in CYP molecules [24]. Considering that the NP size is much larger than the catalytic cavity of CYPs, Lamb et al. [23] and Kulthong et al. [21] have proposed a noncompetitive inhibitory mechanism of these enzymes. In our view, AuNPs likely block the substrate pocket on the CYP surface, depending on the particle characteristics and the unique structural features of the isozymes. This process may represent an additional mechanism for the noncompetitive inhibitory effect of the incubated AuNPs, but the binding domain and the exact residues involved in CYP proteins remain to be confirmed. As one can expect in our observation, the differential sensitivity of these five major CYP isozymes to inhibition by small AuNPs (5 to 20 nm) may be attributed to the structural diversity, heterogeneity, and plasticity among eukaryotic microsomal CYPs [40]. Despite their high sequence similarity shared by all members of this superfamily, these isozymes differ with respect to their active site cavity volumes and drug substrate specificities. The structural analyses of mammalian microsomal CYPs have revealed that the cavity volumes of 1A2, 2D6, 2C9/19, and 3A4 are estimated at around 300, 500, 1,000, and 1,000 to 2,000 Å^3^, respectively [46]. Interestingly, recently reported observations have showed that CYP1A2, which exhibits a much narrower active site cavity than seen for other CYPs, was much less susceptible to inhibition by small NPs [23-25], as was the case in this study for TA-coated AuNPs in human liver microsomes.

## Conclusions

Many types of nanomaterials are reported to have inhibitory effects on different kinds of enzymes including cytochromes P450s. However, comprehensive investigation of the influence caused by metallic nanoparticles on CYP structures and their maker reactions is mostly an unexplored area. Since there is no data available on the relevance of the concentrations used for human exposure, in this mechanistic study, we conduct a systematic investigation of the impacts of AuNPs with different particle sizes and concentrations on five major human CYPs under normal *in vitro* microsomal incubations. It is revealed that AuNPs demonstrate differential inhibitory effects on CYP isozymes in a dose-, size-, time-, and CYP-dependent manner. The strong inhibition of 2C9, 2C19, 2D6, and 3A4 can be anticipated to be a potential source of AuNP-drug interactions for potential nanotoxicological risk assessment when co-exposed with some medicines, as these sensitive CYPs relate to clearance of the largest proportion of drugs and other xenobiotics. We also monitor the time evolution of the dispersion and surface status of AuNPs and characterize their stability and molecular conjugations during incubations. HLM or BSA can perform as a protection layer to prevent against the PBS-induced colloidal nanoaggregation in the AuNP/HLM/PBS mixtures. As expected, the observed red shift of the surface plasmon resonance peak, the drop of surface charge, and the increase in the hydrodynamic diameter further indicate the strong adsorption/interaction between the microsomal components and AuNP surface by incubating a HLM/AuNP complex. Since the nanosized particles may interfere with the CYP functions from the microsomal membrane, the NP-bilayer interactions in vesicular HLMs are important determinants of the overall enzymatic kinetics. Our findings set a basis for investigating NP-related enzyme inhibitions of membrane-bound proteins from the NP-membrane interface. Overall, by combining the activity data of CYP isozymes with direct characterization of AuNPs during the real incubations, we can further explore the potential underpinning mechanisms for controlling the enzyme activities after exposure of TA-coated AuNPs differing in size to the incubation media. Our findings add to recent data suggesting that CYP adsorption to NPs, reversible TA-CYP binding, NP adhesion to HLM membrane, and blocking cavity on the CYP surface may be supported as the major mechanisms for the inhibitory effects caused by AuNPs. Given the inherent heterogeneity and dynamic nature of biomembranes, however, it is difficult to distinguish the propensities for AuNPs to attach onto the CYP protein molecules or disrupt the vesicle HLM membranes. For membrane-bound enzymes, interference from complex nanoparticle-membrane interactions (due to membrane curvature or vesicle rupture) can skew results and complicate the interpretation of the nanoparticle-induced effects on protein functions unless additional steps are taken to remove it. Further studies with complementary experimental approaches and multidimensional *in situ* measurements of the microsome-nanoparticle hybrids will explore a more complete picture of the interaction patterns and molecular dynamics toward a deeper comprehension of the complex NP-induced inhibitory effects. Although physical disruption of microsomal membranes from AuNPs interacting at surface sites cannot be totally eliminated as a possibility, the differential sensitivity of the CYP isozymes to the inhibition suggests that steric blocking effects on the substrate pockets of the isozymes may also offer a plausible explanation to the observed large discrepancy in the inhibitory efficiency. At this point, more work is required to determine the possible and dominant mechanisms of microsome-nanoparticle interaction processes and the extent to which they contribute to *in vivo* nanotoxicology, which will bear importantly upon our understanding of the potential risk and hazard related to nanomaterials.

## Abbreviations

AuNPs: gold nanoparticles; BSA: bovine serum albumin; CE: collision energy; CXP: collision cell exit potentials; CYP: cytochrome P450; DLS: dynamic light scattering; DP: declustering potential; ESI: electrospray ionization; HLM: human liver microsome; IS: internal standard; LC-MS: liquid chromatography-mass spectrometry; MgSO_4_: magnesium sulfate; MRM: multiple reaction-monitoring; NPs: nanoparticles; NaCl: sodium chloride; NADPH: *β*-nicotinamide adenine dinucleotide 2′-phosphate reduced tetrasodium salt hydrate; NaH_2_PO_4_: sodium dihydrogen phosphate; Na_2_HPO_4_: disodium hydrogen phosphate; PBS: phosphate buffer; Q1: precursor ion; Q3: product ion; QC: quality control; RSD: relative standard deviation; SPR: surface plasmon resonance; TA: tannic acid; TEM: transmission electron microscope; UV: ultraviolet.

## Competing interests

The authors declare that they have no competing interests.

## Authors’ contributions

MY carried out the LC-MS assays, CYP inhibition studies, bio-sample preparation and drafted the manuscript. LT performed the characterizations of UV–vis spectroscopy, digital photography, TEM, and DLS measurements. JZ carried out the incubation experiments, results discussion, and analysis data. ML performed nonspecific binding experiments and participated in writing the manuscript. BG conceived of the study, designed, and coordinated the project and drafted the manuscript. YL participated in DLS and TEM measurements. BC participated in design of the study and helped to review the manuscript. All the authors read and approved the final manuscript.

## Supplementary Material

Additional file 1: Figure S1 The optical properties of tannic acid-coated AuNPs. Size-dependent optical properties of tannic acid-coated AuNPs at mass concentration of 1 mg/mL and 25 μg/mL (top); a comparison of the UV/Vis spectra of gold colloid in 6 months of storage (bottom). The UV–vis absorption spectra with the centered bands were consistent with well-dispersed, spherical AuNPs in aqueous media. **Figure S2.** BSA-induced alteration in UV–vis extinction spectra of AuNPs and absorbance value at *λ*_max_ when initial incubating (at 0 min) with different BSA concentrations. **Table S1.** Technical data sheet of PELCO tannic BioPure™ gold nanoparticles. Technical data sheet of physicochemical characteristics of PELCO tannic BioPure™ gold nanoparticles investigated. **Table S2.** Experimental conditions for microsomal CYP assays. Preparation of stock and working solution of substrates, incubation conditions, and analytical parameters for assays. Click here for file
