# Supplementary material for: Size- and time-dependent alteration in metabolic activities of human hepatic cytochrome P450 isozymes by gold nanoparticles via microsomal coincubations
Source: Nanoscale Res Lett. 2014 Nov 28;9(1):642. doi: 10.1186/1556-276X-9-642 (PMC4266508; doi:10.1186/1556-276X-9-642)
Supplement: Additional file 1: Figure S1 — The optical properties of tannic acid-coated AuNPs. Size-dependent optical properties of tannic acid-coated AuNPs at mass concentration of 1 mg/mL and 25 μg/mL (top); a comparison of the UV/Vis spectra of gold colloid in 6 months of storage (bottom). The UV–vis absorption spectra with the centered bands were consistent with well-dispersed, spherical AuNPs in aqueous media. Figure S2. BSA-induced alteration in UV–vis extinction spectra of AuNPs and absorbance value at λmax when initial incubating (at 0 min) with different BSA concentrations. Table S1. Technical data sheet of PELCO tannic BioPure™ gold nanoparticles. Technical data sheet of physicochemical characteristics of PELCO tannic BioPure™ gold nanoparticles investigated. Table S2. Experimental conditions for microsomal CYP assays. Preparation of stock and working solution of substrates, incubation conditions, and analytical parameters for assays. [file 1556-276X-9-642-S1.pdf]

Supporting information for

Size- and time-dependent Alteration in Metabolic Activities of Human Hepatic Cytochrome P450 Isozymes by Gold Nanoparticles via Microsomal Conincubations

Ling Tang,[†] Meiling Ye,[†] Mengjun Luo,[‡] Bin Guo,[*,†] Jing Zhou,[†] Yangyuan Liu,[†] Bo Chen[†]

[†] *Key Laboratory of Chemical Biology and Traditional Chinese Medicine Research (Ministry of Education of China), Key Laboratory of Phytochemical R&D of Hunan Province, Hunan Normal University, Changsha 410081, China*

[‡] *Yiyang Medical College, Yiyang 413000, China*

* *To whom correspondence should be addressed. Tel.: +86 731 85627818; fax: +86 731 85627818. E-mail address: binnguo@126.com (B. Guo)*

Figure S1.

Size-dependent optical properties of tannic acid-coated AuNPs at mass concentration of 1 mg/mL and 25 µg/mL (top); a comparison of the UV/Vis spectra of gold colloid in 6 months of storage (bottom). The UV-Vis absorption spectra with the centered bands were consistent with well-dispersed, spherical AuNPs in aqueous media.

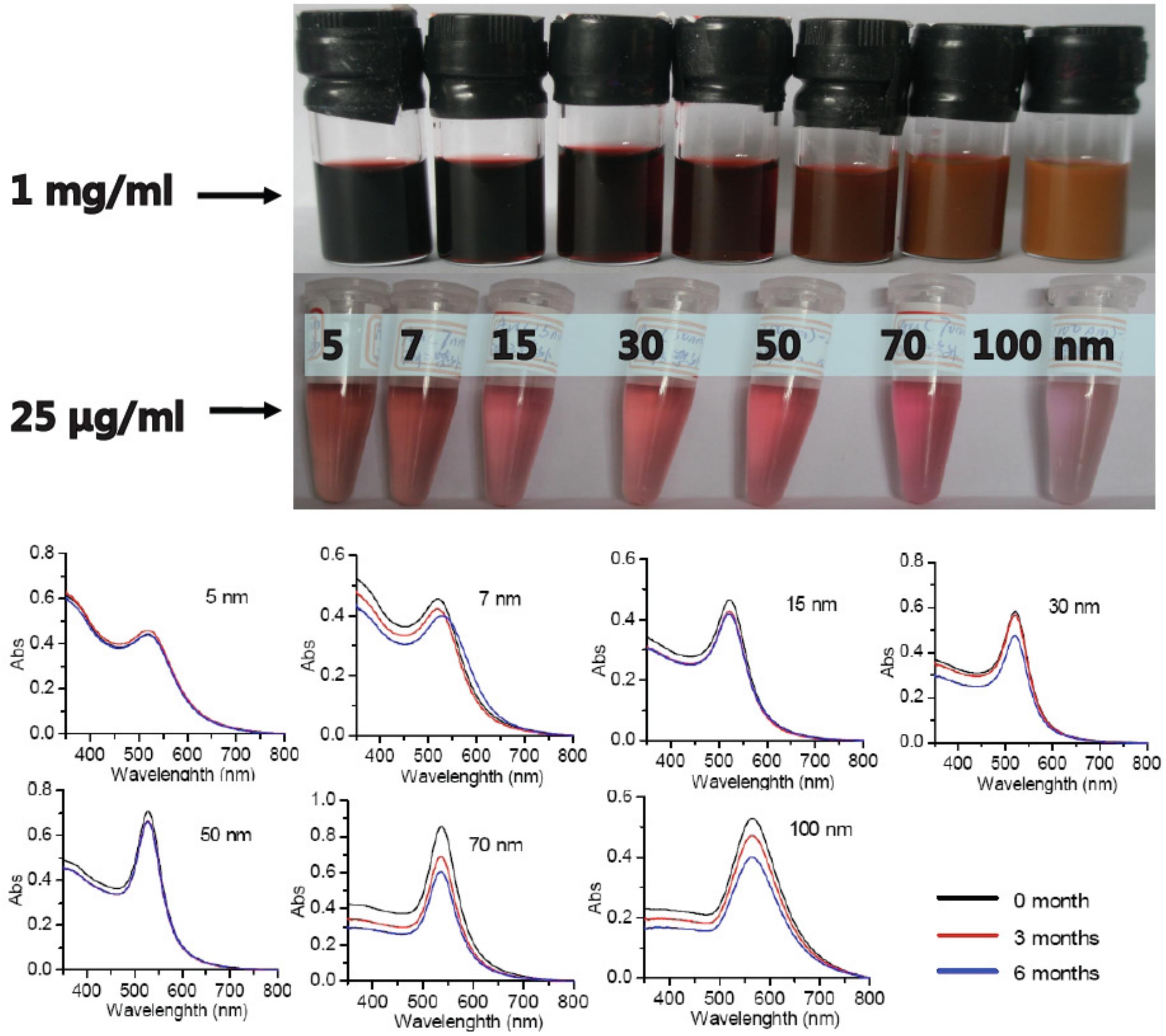

Figure S2.

BSA-induced alteration in UV-vis extinction spectra of AuNPs and absorbance value at λ_{max} when initial incubating (at 0 min) with different BSA concentrations.

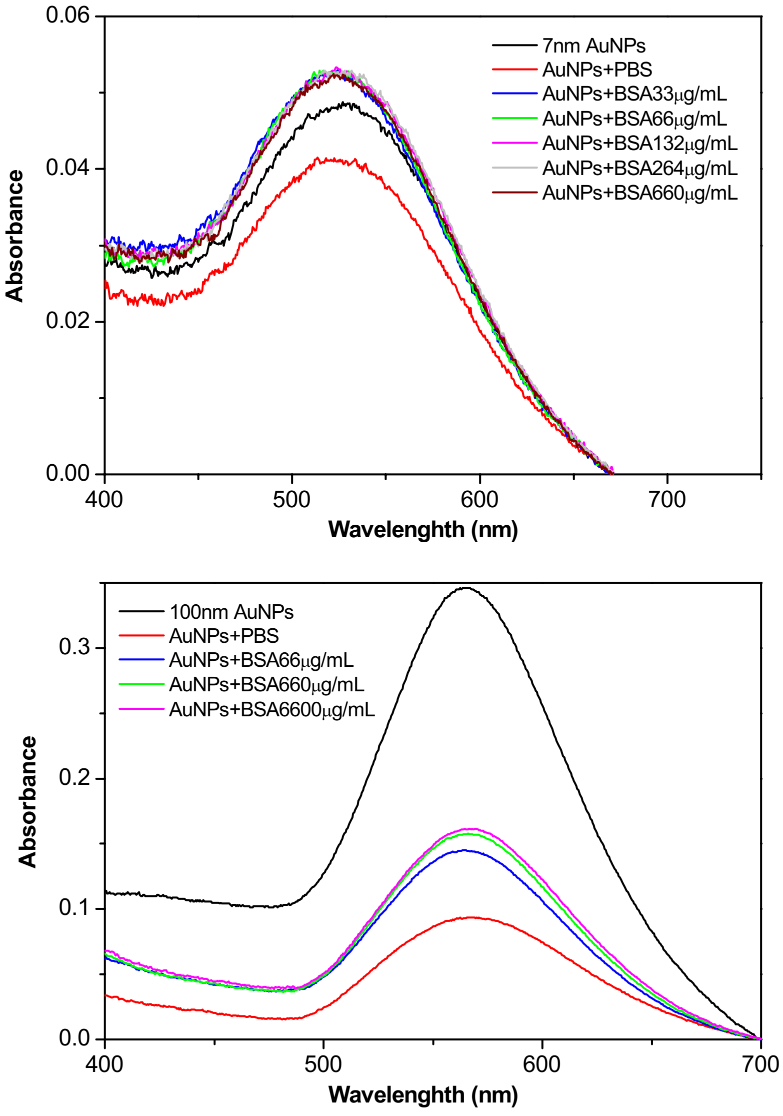

Table S1. Technical data sheet of physicochemical characteristics of PELCO tannic BioPure™ gold nanoparticles investigated

Nominal size	Lot number	Diameter (nm)	Concentration (particles/mL)	Maximum absorption peak (λ_{max}, nm)	Zeta potential (mV)	Hydrodynamic diameter (nm)	Solution pH
5 nm	DAG1519	4.7 ± 0.7	1.1×10^{15}	516	N/A	N/A	4.8
7 nm	DAG1533	6.5 ± 0.9	3.9×10^{14}	519	N/A	N/A	4.6
15 nm	DAG1490	15.5 ± 1.4	2.9×10^{13}	520	N/A	N/A	4.5
30 nm	EAW1056	28.6 ± 3.2	4.3×10^{12}	521	-22.4	34.6	6.4
50 nm	DAG1442	50.1 ± 5.1	7.9×10^{11}	530	-54.9	74.6	7.2
70 nm	DAG1286	67.5 ± 6.2	3.2×10^{11}	538	-43.1	78.2	5.5
100 nm	JMW1048	97.7 ± 9.5	1.1×10^{11}	568	-46.3	109.6	6.2

Table S2. Preparation of stock and working solution of substrates, Incubation conditions and analytical parameters for assays

	Phenacetin *O*-Deethylase (CYP1A2)	Tolbutamide Methylhydroxylase (CYP2C9)	(*S*)-Mephenytoin 4'-Hydroxylase (CYP2C19)	Dextromethorphan *O*-Demethylase (CYP2D6)	Testosterone 6β-Hydroxylase (CYP3A4)	Felodipine Dehydrogenase (CYP3A4)
Preparation of solutions of substrate						
Stock solution (mM) (vehicle)	50.78 (ACN/H_2O=1:1)	60.29 (ACN)	38.95 (ACN/H_2O=1:1)	9.95 (ACN/H_2O=1:1)	21.15 (ACN)	10.41 (ACN)
Working solution (mM) (vehicle)	1.02 (PBS)	2.41 (PBS)	1.61 (PBS)	0.20 (PBS)	1.59 (ACN/H_2O=1:5)	0.10 (H_2O)
Incubation conditions						
Protein concentration (mg/ml)	0.05	0.1	0.15	0.05	0.05	0.05
Subtrates concentration (μM)	5.08	120.58	80.5	9.95	79.67	5.2
Incubation time (min)	10	20	30	10	10	10
Analyte	Acetaminophen	Hydroxy Tolbutamide	4-Hydroxy-mephenytoin	Dextrorphan	6β-Hydroxy-testosterone	Dehydro Felodipine
Mass spectrometer conditions						
Declustering potential (V)	78	72	96	96	98	110
Collision energy (V)	24	18	15	36	20	34
Analyte m/z transition	152.1→110.1	287.2→188.2	235.1→141.3	258.3→199.0	305.3→287.3	383.2→354.1
Retention time (min)	8.04	7.73	7.13	6.23	7.84	11.89
Analytical parameters of IS	m/z transition: 303.5→ 97.3; retention time: 10.50 min; Declustering potential: 106 eV; Collision energy: 37 eV.					
